# Annexin A2 Is a Natural Extrahepatic Inhibitor of the PCSK9-Induced LDL Receptor Degradation

**DOI:** 10.1371/journal.pone.0041865

**Published:** 2012-07-27

**Authors:** Nabil G. Seidah, Steve Poirier, Maxime Denis, Rex Parker, Bowman Miao, Claudio Mapelli, Annik Prat, Hanny Wassef, Jean Davignon, Katherine A. Hajjar, Gaétan Mayer

**Affiliations:** 1 Laboratory of Molecular Cell Biology, Montreal Heart Institute, Département de Médecine and Département de Pharmacologie, Université de Montréal, Montréal, Québec, Canada; 2 Laboratory of Biochemical Neuroendocrinology, Clinical Research Institute of Montreal, Affiliated to the Université de Montréal, Montréal, Québec, Canada; 3 Hyperlipidemia and Atherosclerosis, Clinical Research Institute of Montreal, Affiliated to the Université de Montréal, Montréal, Québec, Canada; 4 Bristol-Myers Squibb Pharmaceutical R & D, Princeton, New Jersey, United States of America; 5 Department of Cell and Developmental Biology, Weill Cornell Medical College, Cornell University, New York, New York, United States of America; University of Milan, Italy

## Abstract

Proprotein convertase subtilisin/kexin-9 (PCSK9) enhances the degradation of hepatic low-density lipoprotein receptor (LDLR). Deletion of PCSK9, and loss-of-function mutants in humans result in lower levels of circulating LDL-cholesterol and a strong protection against coronary heart disease. Accordingly, the quest for PCSK9 inhibitors has major clinical implications. We have previously identified annexin A2 (AnxA2) as an endogenous binding partner and functional inhibitor of PCSK9. Herein, we studied the relevance of AnxA2 in PCSK9 inhibition and lipid metabolism *in vivo*. Plasma analyses of *AnxA2^−/−^* mice revealed: **i)** a ∼1.4-fold increase in LDL-cholesterol without significant changes in VLDLs or HDLs, and **ii)** a ∼2-fold increase in circulating PCSK9 levels. Western blotting and immunohistochemistry of *AnxA2^−/−^* tissues revealed that the LDLR was decreased by ∼50% in extrahepatic tissues, such as adrenals and colon. We also show that AnxA2-derived synthetic peptides block the PCSK9≡LDLR interaction *in vitro*, and adenoviral overexpression of AnxA2 in mouse liver increases LDLR protein levels *in vivo*. These results suggest that AnxA2 acts as an endogenous regulator of LDLR degradation, mostly in extrahepatic tissues. Finally, we identified an AnxA2 coding polymorphism, V98L, that correlates with lower circulating levels of PCSK9 thereby extending our results on the physiological role of AnxA2 in humans.

## Introduction

One of the major causes of death and disability in Western populations is linked to hypercholesterolemia, an important risk factor for atherosclerosis and coronary artery disease (CAD). Hypercholesterolemia affects 1 in 20 subjects and inherited autosomal dominant hypercholesterolemia (ADH; OMIM #143890), which results in even higher levels of cholesterol, occurs at a frequency of 1 in 500 worldwide. Patients affected by ADH are typically characterized by plasma LDL-cholesterol (LDLc) greater that the 95^th^ percentile, presence of tendon xanthomas and premature atherosclerosis. To date, ADH has been linked to heterozygous dominant mutations in the genes encoding the low density lipoprotein receptor (LDLR; 67%), apolipoprotein B (apoB; 14%) or proprotein convertase subtilisin-kexin 9 (PCSK9; ∼2%) [1]. However, ∼17% of ADH-affected patients have no mutations in these 3 loci, indicating that other genes remain to be identified, e.g., on chromosomal cytobands 8q24.22 [2] and 16q22.1 [3].

The discovery of PCSK9, the 9^th^ member of the proprotein convertase family [4], [5], as a third protagonist in ADH [6] has shed light on an unsuspected regulation of LDLR levels in liver [7], [8], [9] and possibly in the brain [10], [11]. PCSK9 undergoes an autocatalytic cleavage of its N-terminal prosegment that remains associated with the catalytic domain [4] and keeps it in an inhibited state [12], [13]. PCSK9 is highly expressed in liver and small intestine [4] and is readily measured by ELISA in plasma [14]. PCSK9 binds the EGF-A domain of the LDLR *via* its catalytic domain [15] and promotes its internalization and degradation in the endosome/lysosome pathway [16], [17], independently of its enzymatic activity [13], [18], [19]. The roles of its N-terminal prosegment and C-terminal Cys/His-rich domain (CHRD) in the subcellular trafficking of the PCSK9≡LDLR complex remain unclear. Deletion of aa 33–58 from the prosegment of PCSK9 results in ∼4-fold enhanced activity on LDLR [20]. However, the CHRD seems to play a critical role in the subcellular trafficking of the cell surface PCSK9≡LDLR complex, since its deletion (aa 456–692) does not prevent PCSK9 binding to LDLR, but abrogates its ability to enhance its degradation [21]. PCSK9 also binds and enhances the degradation of VLDLR and apoER2 [22], [23] that are closely related to LDLR. Indeed, VLDLR proteins accumulate at the cell surface of visceral adipose tissue of *Pcsk9^−^* ^/−^ mice resulting in marked adipocyte hypertrophy [24]. Whether PCSK9 also targets VLDLR in humans is not known [25] and will require deeper analyses of subjects having mutations in the *PCSK9* gene.

The rare gain-of-function (GOF) mutations of PCSK9 identified in ADH-affected patients resulted in a higher ability of PCSK9 to promote LDLR degradation [6], [26]. The strongest one, D374Y increases >10-fold the affinity of PCSK9 for the LDLR and results in very high circulating LDLc (∼10 mmol/L) and early death due to CAD [27]. Loss-of-function (LOF) mutations were also identified, and the 2 nonsense ones Y142X and C679X are particularly frequent (∼2%) in African-Americans [28], [29]. These heterozygote mutations were associated with a ∼40% reduction of LDLc and an 88% reduction in the risk of coronary heart disease [30]. *Pcsk9^−/−^* mouse livers exhibit ∼3-fold more LDLR protein levels and a substantial accumulation of the receptor at the hepatocyte cell surface [7], [9]. This leads to hypocholesterolemia, with a ∼5-fold drop in LDLc levels. In humans, where 70% of cholesterol is associated with LDL, the hypocholesterolemia due to complete PCSK9-deficiency (2 known cases) is even more dramatic (∼85% lower LDLc; 0.4 mmol/L) [31], [32]. This also provided a proof of principle that PCSK9 is a promising and safe target to treat hypercholesterolemia and prevent CAD [33].

Current Canadian guidelines for the prevention and treatment of cardiovascular diseases recommend achieving an LDLc <2 mmol/L (<80 mg/dL) or a 50% reduction in subjects considered at moderate or high risk [34]. Statins, which inhibit the rate-limiting step of cholesterol synthesis catalyzed by hydroxy-methylglutaryl coenzyme A reductase (HMG-CoA reductase), considerably reduced the incidence of atherosclerosis. This cholesterol reduction up-regulates the transcription factor SREBP2, which in turn stimulates the expression of the LDLR resulting in increased LDLc uptake by hepatocytes, and lowering its circulating levels [35], [36], [37]. Statins were shown to reduce cardiovascular events by 25–40% [38]. Statins have an unparalleled safety and efficacy profile, but often lead to suboptimal levels of LDLc in patients with ADH, show variable patient-dependent responses, and/or result in unwanted side effects, emphasizing the need for other molecules to further lower LDLc [39], [40].

In hepatocytes, statins up-regulate PCSK9 mRNA to a greater extent than LDLR [41]. This revealed the paradox that statins on the one hand enhance LDLR level and activity thereby lowering LDLc, but on the other hand increase the expression of PCSK9 that has the ability to destroy the LDLR and oppose its LDL-lowering effect. Therefore, it is believed that neutralization of PCSK9 would enhance the efficacy of statins [7], [42]. Indeed, a significant association of the LOF mutation PCSK9-R46L with statin response was observed in a genome-wide analysis [43]. This supports the hypothesis that the up-regulation of PCSK9 induced by statins attenuates the decrease in LDLc [7], [41], [44], [45]. Lowering PCSK9 levels and/or function has been achieved by antisense mRNA [46], [47], locked nucleic acids [48] and inhibition of PCSK9≡LDLR interaction and degradation using PCSK9 monoclonal antibodies (mAbs) [49], [50], [51], [52], [53]. The latter approach is expensive, restricting it to high risk patients in whom a maximal tolerable dose of statin does not achieve LDLc target levels [34]. Thus, there is a need for cheaper, more accessible inhibitory small molecules, which are not yet available.

Annexin A2 (AnxA2) is strongly expressed in lungs, aorta, heart, adrenals and small intestine [54]. Intracellular AnxA2 is part of a heterotetramer complex comprising two AnxA2 monomers and two copies of its natural binding partner, p11. AnxA2 is composed of an N-terminal 23 aa segment that binds p11, followed by four repeat structures (R1–R4) [55]. Although lacking a signal peptide, AnxA2 is translocated to the cell surface through a p11-dependent mechanism [56] and is found at the cell surface of epithelial [57], [58] and endothelial [59] cells. As a co-receptor for plasminogen and tissue plasminogen activator, which are required for plasmin generation, the AnxA2 complex promotes vascular fibrinolysis. It was suggested that injection of AnxA2 in human may improve thrombotic disease outcome [60]. AnxA2 knockout mice (*AnxA2^−/−^*) are viable, but deficient in endothelial plasminogen processing into plasmin and neoangiogenesis [61].

We previously identified AnxA2 as a natural inhibitor of PCSK9's function on the LDLR, through binding of the first R1 repeat domain of AnxA2 to the CHRD [54]. Inhibition likely occurs *via* an allosteric PCSK9 conformational change induced by AnxA2, similar to what was reported for mAbs that bind the CHRD [62]. In this report, we investigated in more details the molecular interaction of the R1 domain of AnxA2 and PCSK9 and showed that a synthetic peptide spanning the R1 domain is a potent inhibitor. We further hypothesized that PCSK9 is much more active in enhancing LDLR degradation in *AnxA2^−/−^* mice. Indeed, our data showed that *AnxA2^−/−^* mice exhibit a higher level of circulating PCSK9 and lower LDLR protein levels in various tissues previously reported to be refractory to PCSK9's extracellular function on LDLR, such as the adrenals [63]. Furthermore, overexpression of AnxA2 using a recombinant adenovirus resulted in higher LDLR levels in liver. Finally, by sequencing exons of human AnxA2 we identified individuals with a V98L polymorphic variation in the R1 domain, and showed that this variant could be associated with lower levels of circulating PCSK9.

## Materials and Methods

### Expression Constructs

Human PCSK9 with a C-terminal V5 tag and wild type (WT) or mutant AnxA2 cDNAs with a HA-tag were subcloned into pIRES2-EGFP vector (Clontech) and used to perform immunoprecipitation or Far Western blotting, as previously described [54]. All oligonucleotides used for the various AnxA2 constructs are listed in Table S1. All cDNA constructs were verified by DNA sequencing.

### Cell Culture, Transfection, Far Western Blotting and Co-Immunoprecipitation

HepG2, CHO-K1 and HEK293 cells (ATCC) were cultured in DMEM supplemented with 10% fetal bovine serum (Wisent) at 37°C, 95% humidity and 5% CO_2_. Cells (5×10^5^) were seeded in 35 mm dishes and after 24 h, HEK293 cells were transfected with 0.6 µg of plasmid DNA with Effectene transfection reagent (Qiagen) while HepG2 and CHO-K1 cells were transfected with 4 µg of plasmid DNA with Lipofectamine transfection reagent (Invitrogen). Forty-eight hours post-transfection, cells were lysed in ice-cold radioimmune precipitation assay buffer (50 mM Tris-HCl, pH 7.8, 150 mM NaCl, 1% Nonidet P-40, 0.5% sodium deoxycholate, 0.1% SDS) containing a mixture of protease inhibitors (Roche Applied Science). Cell lysates (30 µg) were boiled for 5 min in reducing SDS- polyacrylamide gel electrophoresis (PAGE) sample buffer, separated by SDS-PAGE (8%) and transferred onto nitrocellulose membranes. Far Western blotting was conducted as follows: the nitrocellulose membranes were blocked for 1 h with Tris-buffered saline pH 7.4 containing 0.1% Tween-20 and 1% skimmed milk, and then incubated for 2 h at room temperature with the conditioned media obtained from HEK293 cells overexpressing V5-tagged PCSK9. Bound PCSK9-V5 was then detected with a mouse anti-V5-HRP monoclonal antibody (1∶5000; Invitrogen) and revealed by chemiluminescence using Amersham ECL Plus. The results were recorded by exposure of the membranes to Hyperfilm ECL (GE Healthcare) [54]. Western blotting using a mouse anti-HA-HRP monoclonal antibody (1∶5000; Roche) was performed to verify the expression of AnxA2-HA and mutants thereof. A goat anti-human LDLR (R&D Systems) was used to reveal LDLR in HepG2 cells. For co-immunoprecipitation experiments, CHO-K1 cell lysates were incubated overnight at 4°C with a mouse anti-V5 monoclonal antibody (1∶500; Invitrogen) and 4 h with protein A/G-agarose (Santa-Cruz Biotechnologies). After 5 washes with cold radioimmune precipitation assay buffer, immunoprecipitates were resuspended and boiled for 5 min in reducing SDS-PAGE sample buffer. The presence of WT or V98L HA-tagged AnxA2 was then assessed by Western blot using the anti-HA-HRP monoclonal antibody.

### Media Swap and Immunocytochemistry

Twenty-four hours after transfection, HepG2 cells overexpressing AnxA2 were incubated with conditioned medium of HEK293 cells overexpressing PCSK9-V5. After 60 min at 37°C, cells were fixed in 3.7% paraformaldehyde for 10 min and permeabilized with 0.1% Triton X-100 for 10 min. Non-specific binding sites were blocked with 1% bovine serum albumin and then cells were incubated with mouse monoclonal anti-V5 (1∶500) and goat polyclonal anti-AnxA2 (1∶200; BD Biosciences) antibodies for overnight at 4°C. Following several washes with PBS, antigen-antibody complexes were revealed using mouse and goat specific secondary antibodies coupled to Alexa fluor 555 and Alexa fluor 647 (Invitrogen), respectively. Cells were then covered with 90% glycerol supplemented with 5% 1,4-diazabicyclo[2.2.2]octane (DABCO; Sigma) and examined with an Olympus Fluoview FV10i confocal microscope.

### Animals

WT (Charles River), *AnxA2* ^−/−^ [61] and *Pcsk9^−/−^* [9] mice backcrossed for 7 to 10 times to C57BL/6 mice were housed in a pathogen-free environment in rooms with a 12 h light-dark cycle and fed a chow diet. Male mice of 2–3 months were fasted for 4 h and then anesthetised before collecting blood and tissues. All animal studies were approved by the IRCM Institutional Animal Care and Ethics Committee.

### *In Vivo* Expression of AnxA2 in Mouse Hepatocytes

Recombinant adenoviruses encoding an HA-tagged human AnxA2 cDNA [54] were generated using the Viralpower Expression kit (Invitrogen) as previously described [26]. For *in vivo* expression of AnxA2 in mice, groups of three male mice (2–3 months) were injected intravenously *via* the tail vein with 1×10^11^ particles of recombinant adenovirus on day 0 of the study. Seven-days after injection, mice were fasted 4 h, blood was collected for cholesterol analyses and liver harvested for analyses by Western blotting and immunohistochemistry, as described below. Statistical comparison of data sets was performed by a Student's t-test.

### Plasma Lipids and Fast Protein Liquid Chromatography (FPLC)

For FPLC analyses, individual plasma samples or those pooled from 3 mice (300 µl total) were loaded onto a Superose-6 column with a flow rate of 0.3 mL/min (Pharmacia FPLC System, Amersham Pharmacia Biotech). Fractions of 0.3 ml were collected using an elution buffer (0.01% EDTA, 154 mM NaCl, 0.02% NaN_3_, pH 7.4). Fractions were defined as follows: fractions 15–21, very low-density lipoprotein (VLDL); 22–36, intermediate-density lipoprotein/low-density lipoprotein (IDL/LDL); and 37–55, high-density lipoprotein (HDL). Total plasma cholesterol, triglycerides, free cholesterol, and HDL-C were measured using reagent kits from Wako Chemicals.

### Immunoprecipitation, Immunoblotting and ELISA

PCSK9 was immunoprecipitated from plasma (50 µL) using a rabbit anti-mouse PCSK9 antibody (1∶200) [16] and activated agarose beads coupled with goat anti-rabbit IgGs. Immunoprecipitated proteins were then separated on 8% SDS-PAGE and transferred to a nitrocellulose membrane. Immunoblotting was carried out using the same anti-mouse PCSK9 antibody (1∶3000) and the HRP-conjugated secondary antibody recognizing native IgGs (Trueblot, eBioscience) as previously described [64]. The blots were revealed by chemiluminescence as described above. As loading control, plasma albumin was detected by Western blotting, before immunoprecipitation of PCSK9, using a rabbit anti-mouse albumin (kind gift from Dr. Moïse Bendayan, Université de Montréal). The quantification of mouse plasma PCSK9 level was also assessed by enzyme-linked immunosorbent assay (ELISA) using the CircuLex mouse/rat PCSK9 ELISA (MBL), according to the manufacturer's recommendations.

For tissue immunoblotting, mice were anesthetised and adrenals, liver, ileum and colon were removed and lysed in ice-cold radioimmune precipitation assay buffer supplemented with a mixture of protease inhibitors. Concentrations of protein extracts were estimated by Bradford analysis, and the samples (30 µg/well) were loaded on 8% SDS-PAGE. After protein electro-transfer onto nitrocellulose, membranes were incubated either with goat anti-mouse LDLR (1∶1000; R&D systems), goat anti-AnxA2 (1∶2000; BD Biosciences), anti-HA-HRP (1∶5000; Roche), anti-β-actin (1∶5000; Sigma) or anti-β-tubulin (1∶5000; Sigma) antibodies. The blots were revealed by chemiluminescence as described above using specific HRP-conjugated secondary antibodies. Quantification was performed on scanned film by using the ImageJ (http://imagej.nih.gov/ij/) software (version 1.42). For LDLR quantification, normalization to β-actin or β-tubulin was obtained by calculating the ratio of the level of LDLR to that of β-actin or β-tubulin and by fixing the control ratio to 1. Statistical analyses were performed by Student's t test. A value of p<0.05 was considered significant.

### Immunohistochemistry

Mice were anesthetised and adrenals, liver, ileum and colon were sampled, cold-embedded in Tissue-Tek OCT compound and processed for immunohistochemistry [65]. Seven-µm-thick cryosections were mounted on glass slides, fixed in acetone∶methanol (1∶1) at −20°C for 2 min and washed in PBS at room temperature. Fixed cryosections were incubated with goat anti-mouse LDLR (1∶100, R&D systems; [9]), goat anti-AnxA2 (1∶25, BD Biosciences) or rabbit anti-HA (1∶100, Sigma) antibodies overnight at 4°C. Sections were then washed 4 times in PBS and incubated with Alexa 488- or Alexa 555-conjugated secondary antibodies raised in donkey (Invitrogen) for 1 h. Then, cryosections were washed again several times in PBS and nuclei were stained with the TO-PRO-3 DNA dye (Invitrogen). Sections were mounted in 90% glycerol supplemented with 5% 1,4-diazabicyclo[2.2.2]octane (DABCO; Sigma) and examined with a Zeiss 510 confocal microscope. Negative controls of immunolabelling were performed by omission of the primary antibodies.

### Quantitative Real Time PCR

Quantitative real time PCR analysis was performed as previously described [54]. Briefly, RNA from tissues of 4 to 6 mice was extracted using Trizol reagent (Invitrogen). After reverse transcription, each cDNA sample (in triplicate) was submitted to two PCR amplifications; one for the mouse ribosomal-S16 gene used as a normalizer (forward primer GCTACCAGGGCCTTTGAGATG and reverse primer AGGAGCGATTTGCTGGTGTGG) and the other for *Ldlr* (forward primer GTATGAGGTTCCTGTCCATC and reverse primer CCTCTGTGGTCTTCTGGTAG), *Pcsk9* (forward primer TGCAAAATCAAGGAGCATGGG and reverse primer CAGGGAGCACATTGCATCC), *AnxA2* (forward primer GATTAGAATCATGGTCTCTCG and reverse primer TTAGTGGAGAGCGAAGTCTC), *Hmg-CoA* reductase (forward primer GTACGGAGAAAGCACTGCTGAA and reverse primer TGACTGCCAGAATCTGCATGTC) or *Srebp-2* (forward primer GTTCTGGAGACCATGGAG and reverse primer AAACAAATCAGGGAACTCTC) genes. The Mx3500P system from Stratagene was used to perform and analyze the quantitative real time PCRs.

### Solid Phase Synthesis of AnxA2 Peptides

Peptides were prepared by solid phase synthesis using a Liberty microwave peptide synthesizer (CEM Corp.). The Fmoc deprotection and coupling steps were performed at 75°C using microwave heating with pulsing sequences of 20 W with reaction temperatures monitored by fiberoptic probe. A PAL-derivatized solid support (PCAS BioMatrix Inc.) was utilized to generate C-terminal peptide amides from the peptide cleavages. The amino acids were coupled in 4- to 5-fold excess using methods provided by the manufacturer and as appropriately modified. At the beginning of each coupling step, the Fmoc group was removed by two 5 min treatments with 5% piperazine in dimethylformamide (DMF) containing 0.1 M N-hydroxybenzotriazole. After 5 DMF washes, the desired Fmoc-amino acids were successively single coupled by activation with 0.5 M 2-(6-Chloro-1-H-benzotriazol-1-yl)-1,1,3,3-tetramethyluronium hexafluorophosphate in DMF and 2 M Diisopropylethylamine in N-methylpyrrolidone for 5 minutes. At the end of each coupling, the resin was washed with DMF 5 times. Upon completion of the sequence assembly, the N-terminus of the peptidyl-resin was acetylated using a solution of 10% Acetic Anhydride and 1% Diisopropylethylamine in DMF. After the capping step, the peptidyl-resin was washed with dichloromethane and dried in vacuum. The peptides were then released from resins and concomitantly deprotected by treatment with trifluoroacetic acid (TFA)/water/triisopropylsilane (94∶3∶3; v∶v∶v; 45 mL/g of resin) for 2 hrs at 20°C. The spent resin was filtered off and rinsed with additional cleavage solution, the combined filtrates were evaporated to 1/10 volume, and the crude peptide product was precipitated by addition of diethyl ether. The precipitated product was collected by centrifugation, washed with additional ether, and dried to yield the peptide products as off-white solids.

The peptides were then purified by preparative reverse phase high performance liquid chromatography (RP-HPLC) by injection into a Phenomenex C18 column (21.2×250 mm; 5°C) and elution using gradients of 0.1% TFA in acetonitrile against 0.1% TFA in water. The fractions containing clean product (as determined by analytical HPLC) were combined and lyophilized to yield purified peptides with >90% purity. The correct identity of each peptide was determined by high performance liquid chromatography/mass spectrometry analysis in electrospray mode. In each case, the experimental molecular weight derived from the multiply charged m/z ions was within 1.0 Daltons of the calculated molecular weight.

### Alphascreen Assay for PCSK9≡LDLR

The Amplified Luminescent Proximity Homogeneous Assay (AlphaScreen) technology provides a non-radioactive assay for the detection of protein-protein interactions. An AlphaScreen was developed to investigate the interaction between PCSK9 and LDLR. Purified recombinant human LDLR ectodomain protein (residues 1–699, his-tagged) and biotin-labeled human PCSK9 protein (residues 30–692) were produced by baculovirus expression as described previously [20]. For the assay, PCSK9 (15 nM) and LDLR (15 nM) were mixed and the AnxA2 test peptide was added as indicated in figure legend, and incubated for 60 min at 20°C in buffer (25 mM Hepes, pH 7.4, 100 mM NaCl, 0.5 mM CaCl_2_, 0.1% BSA). Streptavidin donor beads and nickel chelate acceptor beads (Perkin-Elmer) were mixed at 1∶1 ratio and added to wells at 20 µg/mL each. The assay mixture was incubated overnight at 20°C, and the AlphaScreen luminescence signal was measured by a Perkin Elmer Envision instrument at 580 nm emission wavelength.

### Human Subjects, Sample Handling, and Sequencing of AnxA2 Single Nucleotide Polymorphisms

All subjects gave informed written consent, and the IRCM ethics committee approved the protocol. Plasma samples and blood leukocytes were taken from a cohort of 74 volunteers and patients over 18 years of age, as described [14]. The physical characteristics, fasting plasma lipids, and PCSK9 levels are shown in Table S2. Total and lipoprotein cholesterol were quantified at the laboratory of the Centre Hospitalier de l'Université de Montréal using a standard enzymatic method. LDLc was calculated using the Friedewald equation and plasma PCSK9 concentration was quantified by ELISA as described [14]. DNA was extracted from white blood cells using QIAmp Blood Maxi kit (Qiagen) according to the manufacturer's instructions. The sequences of the primers used for amplifying exons 4, 5, 6 of AnxA2 were obtained from NCBI at http://www.ncbi.nlm.nih.gov/genome/probe/ using the resequencing amplicons for AnxA2. For the detection of human AnxA2 SNPs occurring in the R1-domain, we followed the VariantSeqr™ (Applied Biosystems) protocol. Resequencing amplicons (RSA) for exons 4, 5 and 6 coding for R1-domain aa 37–108 were produced from genomic DNA preparations by PCR using the following specific primer pairs; Exon 4, RSA probe RSA001290881, resequencing amplicon (636 bp) M13-Forward primer GTAAAACGACGGCCAGTGCATGGGTTGAGCTAGGTCTGGA, M13-Reverse primer CAGGAAACAGCTATGACCTTCGAATTGTTGCCAGCCCA, Exon 5, RSA probe RSA000545091, resequencing amplicon (567 bp) M13-Forward primer TGTAAAACGACGGCCAGTTGTGAACTGCACACGCGAGG, M13-Reverse primer CAGGAAACAGCTATGACCCATTGGGTAGAGGATGCTGACGA, Exon 6, RSA probe RSA000059987, resequencing amplicon (556 bp) M13-Forward primer TGTAAAACGACGGCCAGTTTCCAATTGCCCAGGTGCTG, M13-Reverse primer CAGGAAACAGCTATGACCGGGAATGCAGTTGAGGGCGT. The amplified fragments were purified from agarose gels using QIAquick Gel Extraction kit (Qiagen), treated with ExoSAP-It (USB corp.) and sequenced on a 3130 XL Genetic analyzer from ABI (Applied Biosystems) using universal M13 Forward and Reverse sequencing primers. Sequences were analyzed with SequencherTM software (Gene Codes Corporation).

## Results

### *AnxA2^−/−^* mice have higher levels of circulating LDLc and PCSK9

Our previous study on AnxA2 inhibition of PCSK9's activity towards the LDLR [54] led us to hypothesize that plasma cholesterol would be affected by the absence of *AnxA2 in vivo*. To determine if AnxA2 plays a role in cholesterol homeostasis, we first analyzed the plasma lipid profiles of *AnxA2^−/−^* mice. FPLC analyses of plasma from 6 *AnxA2^−/−^* and 5 WT mice revealed that LDLc levels (combined IDL/LDL fractions) were significantly increased by ∼40% in the absence of AnxA2, while VLDLc, HDLc, triglycerides and total cholesterol levels were not significantly modified (Table 1, Figure S1). These results demonstrate that loss of AnxA2 is associated with higher levels of circulating LDLc, possibly through the lack of inhibition of PCSK9.

**Table 1:** Comparison of plasma lipid profiles of wild-type C57BL/6 mice versus *AnxA2*−/− mice by FPLC.

	C57BL/6 (n = 5)	AnxA2 KO (n = 6)	Fold change
Total cholesterol (mg/dL)	80.4±8.0	82.7±3.0	X 1.03
IDL/LDL cholesterol (mg/dL)	13.9±2.0	**19.6±2.0**	**X 1.41** *
VLDL cholesterol (mg/dL)	3.8±0.6	3.7±1.5	X 0.97
HDL cholesterol (mg/dL)	61.6±6.1	59.4±2.1	X 0.96
Triglycerides (mg/dL)	30.8±11.4	23.0±15.1	X 0.75

Values are mean ± SD.

Significant differences from wild-type mice are indicated as:

* , p<0.01.

**Figure 1:**
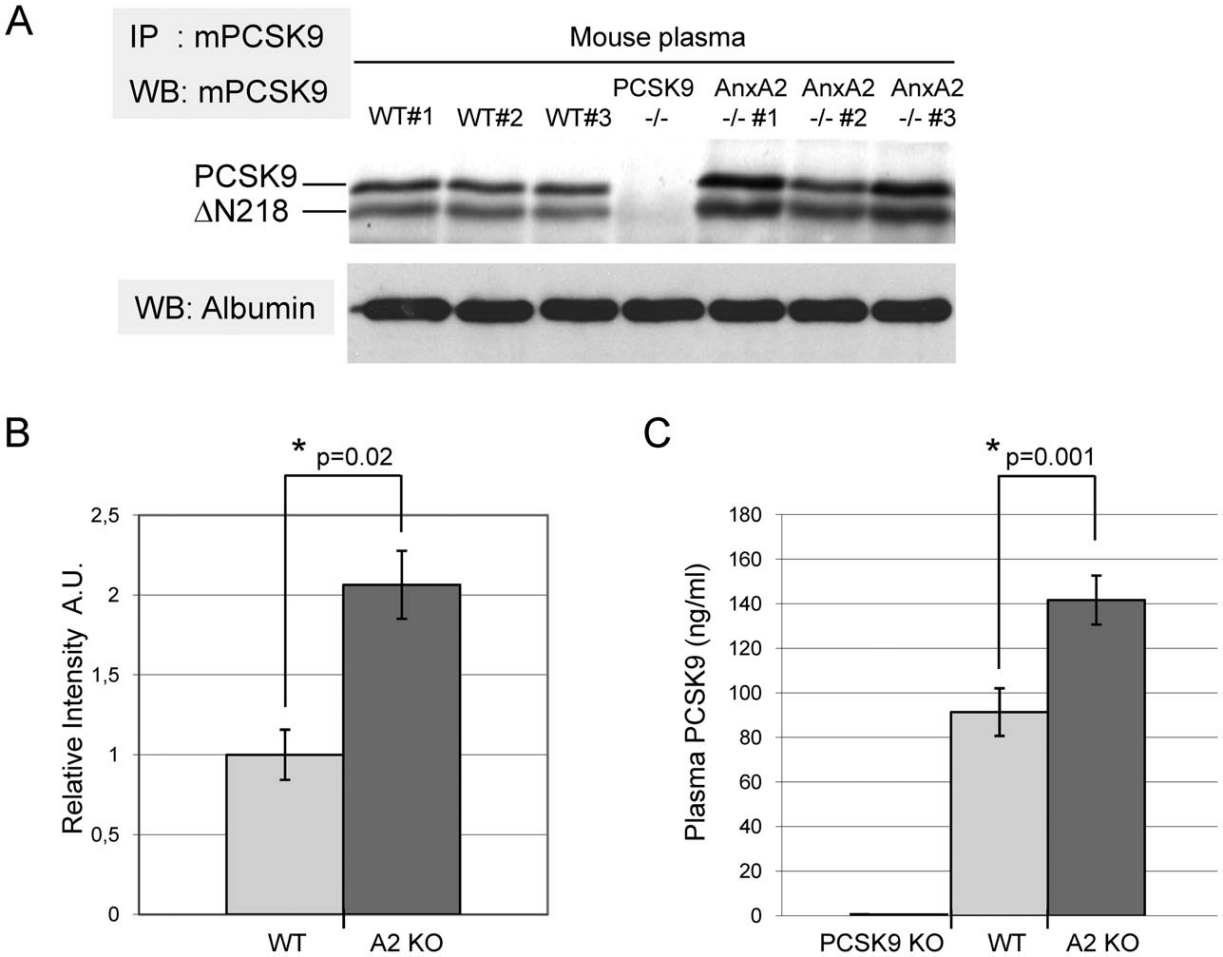
Circulating PCSK9 is increased in *AnxA2^−/−^* mice. (A) PCSK9 in plasma samples from wild-type (WT) and *AnxA2* ^−/−^ mice was immunoprecipitated (IP) using an anti-mouse PCSK9 (mPCSK9) polyclonal antibody and detected by Western blotting (WB) using the same antibody as described in Materials and Methods. Mature full-length PCSK9 and its furin-cleaved form (ΔN218) are detected in WT and *AnxA2* ^−/−^ mice. As control, the same method was used to immunoprecipitate PCSK9 in plasma of *PCSK9* ^−/−^ mice and resulted in the absence of bands. As loading control, plasma albumin was detected by Western blotting, before immunoprecipitation of PCSK9, using a rabbit anti-mouse albumin. (B) Western blots of immunoprecipitated plasma PCSK9, as shown in (A), were quantified by scanning densitometry (n = 7 WT mice plasma samples and n = 8 *AnxA2* ^−/−^ mice plasma samples). (C) Quantification of plasma PCSK9 level from WT and *AnxA2* ^−/−^ mice using an ELISA assay (n = 1 *Pcsk9* ^−/−^, n = 4 WT and n = 3 *AnxA2* ^−/−^ mice plasma samples). Bars and error bars represent average ± SD. P-values were obtained using a two-tailed Student's t-test.

AnxA2 levels are low in hepatocytes where PCSK9 is highly expressed (Figure S2) and is the most active at inducing LDLR degradation [63], [66], [67]. However, cell surface AnxA2 lining blood vessels could bind and sequester circulating PCSK9 that is secreted from hepatocytes [24], thereby decreasing its effect on LDLR. One possible consequence resulting from such binding would be an increase of circulating PCSK9 in mice lacking AnxA2. Therefore, equal volumes of plasma samples (also demonstrated by equivalent albumin levels, Figure 1) from male WT and *AnxA2^−/−^* mice were immunoprecipitated with a specific polyclonal anti-mouse PCSK9 antibody [16]. Circulating PCSK9 levels were significantly increased by ∼2-fold in the plasma of *AnxA2^−/−^* mice when compared to WT mice (Figure 1A,B). Quantification of plasma PCSK9 levels by ELISA also revealed an increase by ∼1.6-fold (Figure 1C). No differences were detected in the ratio of full-length PCSK9 and its furin-cleaved form PCSK9-ΔN_218_ [68], [69] and both were increased compared to WT mice (Figure 1A). Thus, furin cleavage of PCSK9 at the cell surface of hepatocytes [70], [71], [72] precedes the binding of these two circulating CHRD-containing forms to AnxA2. This doubling of PCSK9 levels in plasma could contribute to the observed ∼40% increase in circulating LDLc levels seen in *AnxA2^−/−^* mice (Table 1, Figure S1) possibly through enhanced LDLR degradation.

### Reduction of LDLR Levels in Extrahepatic Tissues of *AnxA2^−/−^* Mice

**Figure 2:**
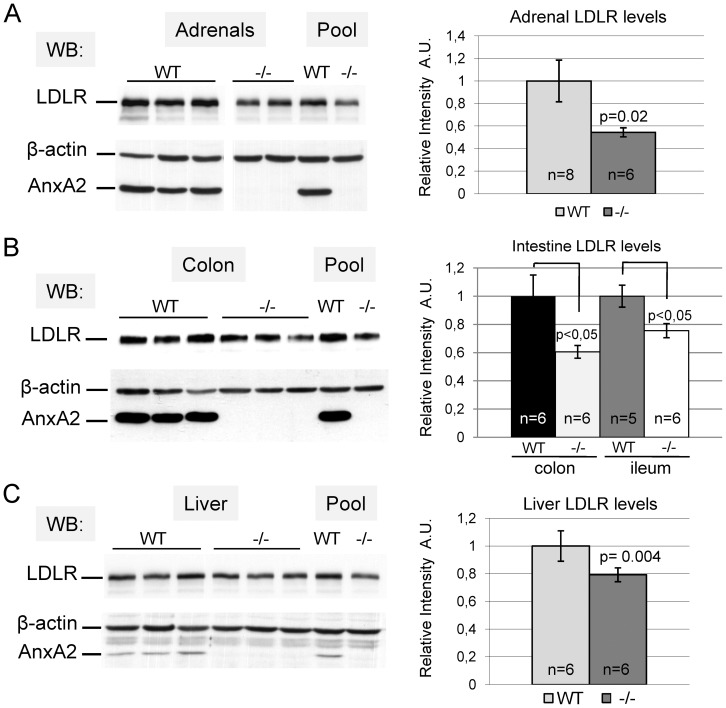
LDLR level is decreased in extrahepatic tissues of *AnxA2* ^−/−^ mice. (A–C) Western blots of LDLR and AnxA2 in individual tissue samples or tissue pools for adrenals (A), ileum, colon (B) and liver (C) of WT and *AnxA2* ^−/−^ mice. Scanning densitometry quantification of WB signals obtained for LDLR is shown on the right panels in A–C. LDLR relative intensity was normalized over the signal obtained for β-actin. For each genotype, the number of animals used for quantification is indicated (n = 5–8 per group). Bars and error bars represent average ± SD. P-values were obtained using a two-tailed Student's t-test.

**Figure 3:**
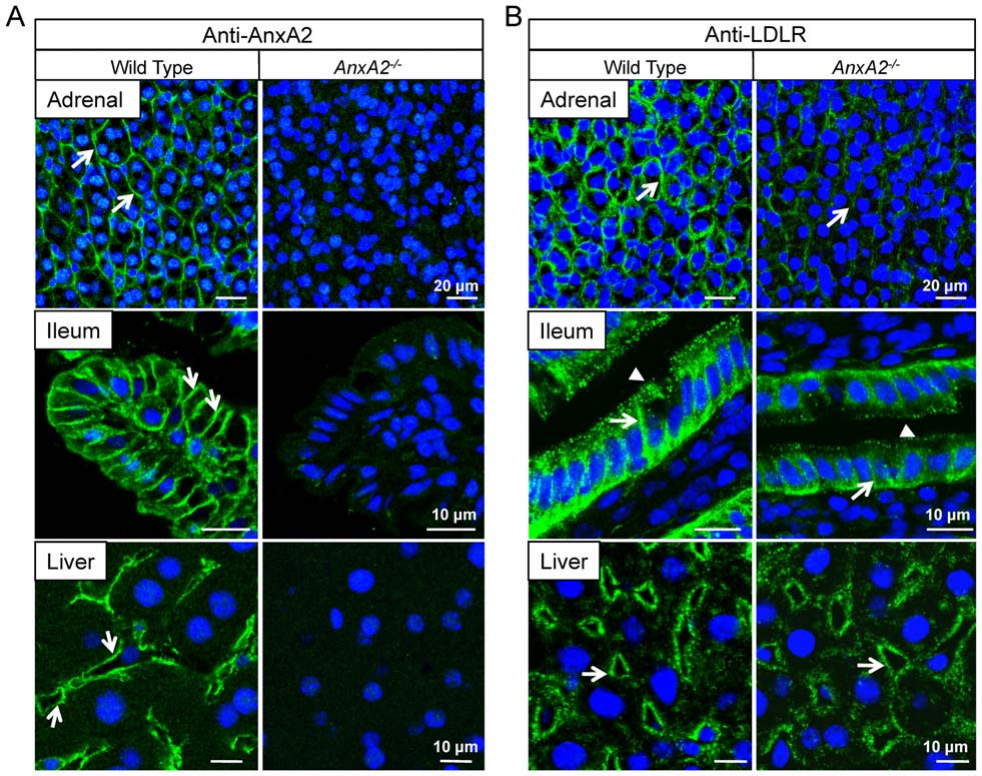
Immunofluorescence staining of LDLR and AnxA2 in tissues of WT and *AnxA2* ^−/−^ mice. Frozen adrenal, ileum or liver tissue sections were fixed and incubated with anti-AnxA2 (A) or anti-LDLR (B) antibodies. Bound primary antibodies were revealed with species-specific Alexa-488 (green) secondary antibodies. Nuclei were counterstained with the blue fluorescent DNA dye DAPI. (A) Arrows in left panels depict the localization of AnxA2 along capillaries of the adrenal cortex (top panel), at the basolateral membrane of absorptive cells in the ileum (middle panel) and in capillaries of sinusoids in the liver (bottom panel) of WT mice. The absence of AnxA2 labelling in tissues of AnxA2^−/−^ mice (right panels) demonstrates the specificity of the antibody. (B) Arrows in left panels depict the localization of LDLR at the cell surface of adrenal cortical cells (top panel), at the basolateral membrane and apical surface (arrowheads) of absorptive cells in the ileum (middle panel), and at the basolateral surface of hepatocytes facing sinusoids (bottom panel) of WT mice. The corresponding LDLR staining of *AnxA2^−/−^* mice tissues is significantly decreased in the adrenal and ileum while it shows less variation in the liver (right panels).

Adrenal glands strongly express both AnxA2 and LDLR but very low levels of PCSK9 mRNAs (Figure 2A, Figure S2). QPCR analysis did not reveal a significant change in the mRNA levels of either PCSK9 or LDLR in *AnxA2^−/−^* liver, adrenals, ileum or colon (*not shown*). In adrenal glands of *AnxA2^−/−^* mice, LDLR protein levels were decreased by ∼50% (Figure 2A), suggesting increased LDLR degradation in the absence of AnxA2. By immunohistochemistry, AnxA2 was localized in the adrenal cortex, where staining appears to reside mostly in capillary endothelial cells lining cords of glandular epithelial cells (Figure 3A, upper panels). The specificity of the antibody is demonstrated by the lack of labelling in the adrenals of *AnxA2^−/−^* mice (Figure 3A) and by the absence of AnxA2 immunoreactivity on Western blots of *AnxA2^−/−^* adrenals (Figure 2A). The intensity of LDLR staining, which is present at the cell surface of adrenal cortical cells in WT mice, was greatly decreased in *AnxA2^−/−^* mice (Figure 3B, upper panels), supporting the Western blot data. Since the expression of PCSK9 is very low in adrenals, this decrease of LDLR may be the consequence of the ∼2-fold higher levels of circulating PCSK9 in *AnxA2^−/−^* mice (Figure 1). LDLR levels were also measured in the ileum and colon that express high levels of LDLR and AnxA2 mRNA, but also PCSK9, albeit at ∼6-fold lower levels than the liver (Figure S2). Western blots of tissue lysates demonstrated that the LDLR was reduced by ∼40% in the colon and by ∼25% in the ileum of *AnxA2^−/−^* mice (Figure 2B). The difference in LDLR levels in the ileum versus the colon, in absence of AnxA2, could arise from the differential expression of endogenous PCSK9 or the different accessibility of endogenous and circulating forms of PCSK9 to the LDLR. In addition, the putative membrane-bound protein responsible for the targeting of the PCSK9≡LDLR complex to lysosomes for degradation [16], [21] has not been identified nor its sensitivity to AnxA2, since both proteins are predicted to bind the C-terminal CHRD of PCSK9 [54]. The immunohistochemical staining showed that both AnxA2 and LDLR were localized at the basolateral membrane of the intestinal absorptive cells, while only the LDLR was found over the apical brush border (Figure 3A and 3B, middle panels). In agreement with Western blotting data, LDLR staining was significantly decreased in the ileum (Figure 3B, middle panels) and colon (*not shown*) of *AnxA2^−/−^* mice. In the liver, the AnxA2 mRNA is hardly expressed (Figure S2), which is also reflected by the weak signal detected for its protein product by Western blot (Figure 2C). AnxA2 was localized in endothelial cells of sinusoids, while almost no staining was present in hepatocytes (Figure 3A, bottom panels). By contrast, the LDLR was localized at the basal surface of hepatocytes facing the sinusoids (Figure 3B, bottom panels). In the *AnxA2^−/−^* liver, the LDLR staining intensity showed little difference compared to WT mice (Figure 3B, bottom panels), although the total LDLR protein level was decreased by ∼20% (Figure 2C). Taken together, these results suggest a role of AnxA2 in regulating mostly extrahepatic LDLR levels and plasma LDLc through the inhibition/capture of circulating PCSK9.

Interestingly, in media swap experiments where HepG2 cells overexpressing AnxA2 were incubated with conditioned medium of HEK293 cells overexpressing PCSK9-V5, we found that PCSK9 could be internalized and co-localized with AnxA2 in intracellular compartments (Figure S3). Compared to surrounding untransfected cells where PCSK9 appeared in small endosomal-like vesicles, PCSK9 was found in larger endosomal structures possibly indicating an accumulation or a slower trafficking toward lysosomes. In this context, we can speculate that circulating PCSK9 could bind AnxA2 at the plasma membrane and also in intracellular compartments of extrahepatic organs. The amount of cell surface AnxA2 could dictate how much PCSK9 is available to degrade LDLR in a given organ. Indeed, it was shown previously that AnxA2 could compete with the LDLR for binding to PCSK9 [54].

### Identification of Critical PCSK9 Binding Residues in the R1 Domain of AnxA2

We previously reported that AnxA2 binds PCSK9 by its R1 domain [54]. Herein, we deleted stretches of the R1 domain starting from the N-terminal Gly^25^ and proceeded to binding assays by far Western blotting (Figure S4A,B). HEK293 cells were transiently transfected with human HA-tagged AnxA2 deletants (Δ25–36, Δ37–48, Δ49–61, Δ62–75, Δ37–66, Δ74–88, Δ82–88, Δ89–101, Δ102–108) and 48 h post-transfection, cell lysates were subjected to (**i**) Western blotting with the anti-HA-HRP antibody to evaluate the expression of AnxA2 mutants, and to (**ii**) far Western blotting to assess PCSK9 binding to AnxA2 [54]. As shown in Figure S4B, all deletants prevented PCSK9 binding. By taking advantage of the high sequence homology between AnxA1 and AnxA2 (Figure S4A,C) and the fact that PCSK9 does not interact with AnxA1 [54], we decided to sequentially interchange blocks of three aa from the corresponding R1 domain of AnxA1 into AnxA2 (AnxA1>AnxA2; Figure S4A,D). Far Western blotting revealed that PCSK9 binding to AnxA2 does not require aa 25–33 and that the critical segment begins at aa 34, a position corresponding to the beginning of the first α-helix (aa 34–48; Figure S4C,D). In addition, we noticed that swapping AnxA1 aa 40–42, 43–45 or 46–48 into AnxA2 only moderately affects PCSK9 binding, suggesting that the N-terminal residues of the first α-helix (aa 34–39) of AnxA2 are critical for its interaction. Surprisingly, the interchange of the highly conserved sequence aa 49–75 of AnxA1 and AnxA2, comprising an exposed loop (aa 49–53) and two α-helices buried in the core of AnxA2, significantly affected PCSK9 binding (Figure S4C,D). Moreover, we previously showed that the C-terminal charged residues ^77^ RRTKK ^81^ in the R1 domain were critical for PCSK9 binding [54]. Therefore, we also investigated the importance of the N-terminal charged residues ^28^K, ^34^D, ^36^E, ^37^R, ^43^E and ^47^K in AnxA2. For this purpose, rather than substituting them with a neutral Ala, we replaced them with the equivalent residues found in AnxA1 in groups of three, i.e., K28S+D34N+E36S and R37S+E43H+K47M. The Far Western data suggest that aa ^37^R, ^43^E, ^47^K are critical for PCSK9≡AnxA2 interaction, whereas ^28^K, ^34^D, ^36^E moderately participate in PCSK9 binding (Figure S4C,D). Thus, the complex interaction of AnxA2 with PCSK9 involves the entire R1 domain aa 34–108, but most likely depends on clusters of exposed charged residues together with the coiled-coil patterns found in the AnxA2 α-helices.

### AnxA2 inhibition of the PCSK9≡LDLR interaction *in vitro*

**Figure 4:**
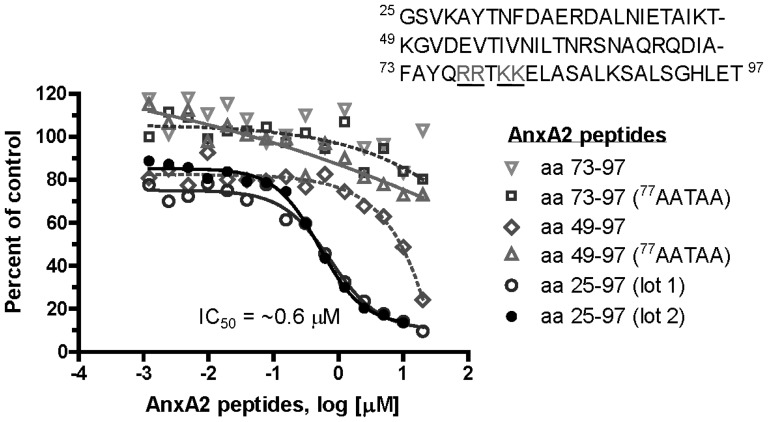
Peptides of AnxA2 R1 domain interfere with the PCSK9≡LDLR interaction. His-tagged LDLR ectodomain (15 nM) and biotinylated PCSK9 (15 nM) were mixed and incubated in the presence of increasing concentration of AnxA2 peptides for 60 min at 20°C. Streptavidin donor beads and nickel chelate acceptor beads were added to the assay mixture and incubated overnight at 20°C. The AlphaScreen luminescence signal was measured at 580 nm emission wavelength. The competition of AnxA2 peptides with the PCSK9≡LDLR interaction was revealed by a decrease in luminescence signal and demonstrated that the longest AnxA2 peptide (aa 25–97) inhibits binding of PCSK9 to LDLR with an IC_50_ of 0.6 µM. ^77^AATAA refers to the mutation of ^77^ RRTKK ^81^.

Based on the far Western data, we synthesized a 73 aa peptide corresponding to the AnxA2 R1 domain (residues 25–97), as well as intermediate 49 aa (residues 49–97) and small 25 aa (residues 73–97) forms. Using an AlphaScreen assay to measure PCSK9 protein∶protein interactions with the soluble LDLR ectodomain, we found that the long peptide form was the most potent inhibitor of PCSK9 binding to the LDLR. The 73-mer AnxA2 peptide (aa 25–97) exhibited an IC_50_ of ∼0.6 µM in this assay; and similar results were seen with 2 different lots of the peptide (Figure 4). The intermediate length peptide (49–97) was active but with ∼20-fold lower apparent potency, while the short form (73–97) was inactive in this assay (Figure 4). In agreement with our previous observation that the C-terminal charged residues ^77^ RRTKK ^81^ are critical for PCSK9 binding to AnxA2 [54], the intermediate length peptide incorporating 4 alanine substitutions (^77^AATAA^81^) lost its activity entirely (Figure 4). The data suggest that AnxA2 R1 domain peptides specifically bind full length PCSK9 and inhibit its interaction with LDLR, consistent with the activity seen for the full length AnxA2 protein. Since PCSK9 lacking its CHRD does not bind AnxA2 [54], we only concentrated on the full length PCSK9 to identify the best inhibitory peptide that would interfere with its interaction with the LDLR. AnxA2 R1 domain peptides represent useful starting points as tools to evaluate pharmacological inhibition of PCSK9 function.

### Adenoviral Expression of AnxA2 in Mice Increases Hepatic LDLR Levels

**Figure 5:**
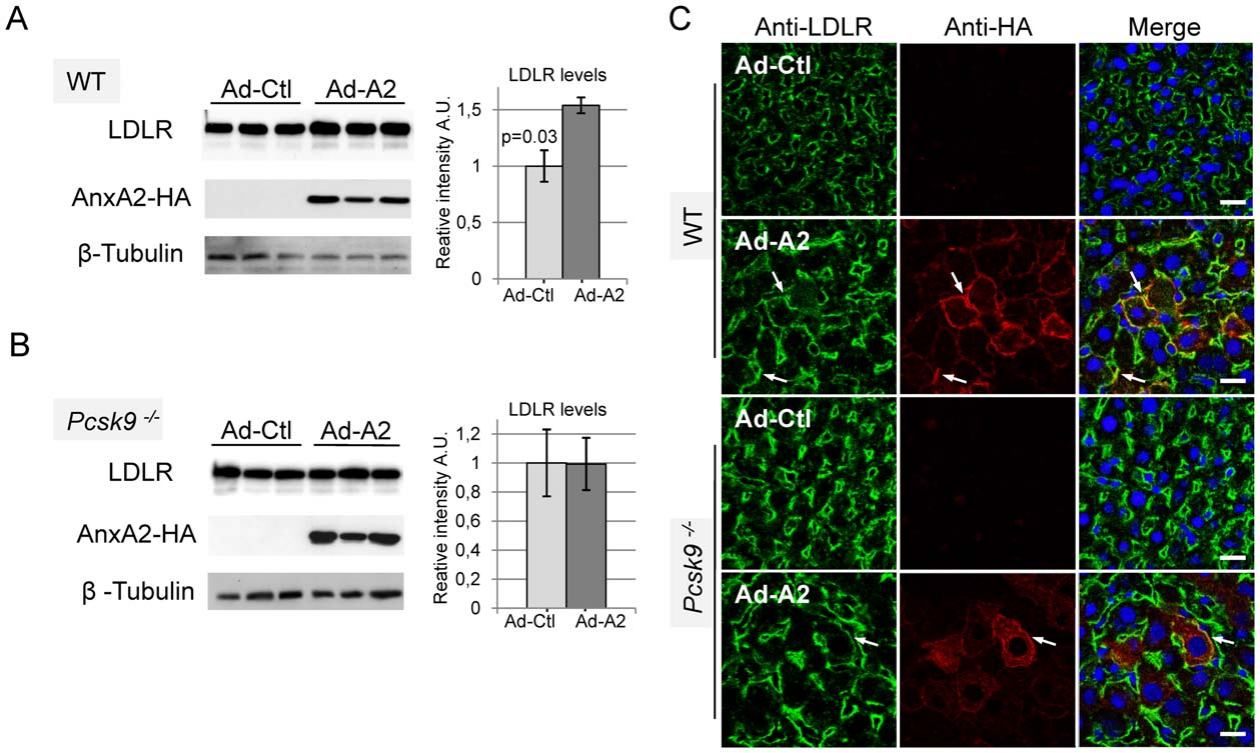
Adenoviral overexpression of AnxA2 in mouse liver significantly increases LDLR levels. Empty control (Ad-Ctl) and HA-tagged AnxA2 (Ad-A2) adenoviruses were injected intravenously into the tail vein of WT (A) or *Pcsk9^−/−^* (B) mice. After 7 days, livers were analysed for LDLR levels and AnxA2-HA expression by Western blotting. Bars and error bars represent average ± SD. P-values were obtained using a two-tailed Student's t-test. (C) Immunohistochemistry of LDLR and AnxA2-HA in the liver of WT (upper panels) or *Pcsk9^−/−^* (lower panels) mice injected with Ad-Ctl or Ad-A2 adenoviruses. Frozen liver tissue sections were fixed and incubated with anti-LDLR and anti-HA antibodies. Bound primary antibodies were revealed with species-specific Alexa-488 (LDLR, green) and Alexa-555 (HA, red) secondary antibodies. Nuclei were counterstained with DAPI (blue). Arrows show colocalization of LDLR with AnxA2-HA at the cell surface of hepatocytes. Bars = 20 µm.

In order to test if AnxA2 could inhibit PCSK9 and increase LDLR protein levels *in vivo*, we used a recombinant adenovirus-mediated gene transfer technique to direct AnxA2 to the liver of mice. Eight- to ten-week old C57BL/6 WT or *Pcsk9^−/−^* mice fed on chow diet were tail vein injected with an adenovirus vector encoding HA-tagged human AnxA2 (Ad-A2), or an empty adenovirus vector (Ad-Ctl) used as a control (10^11^ particles per mouse, 3 mice for each group). Seven days post-infection, the liver was dissected and AnxA2-HA overexpression was confirmed by Western blotting (Figure 5A,B) and immunocytochemistry (Figure 5C, middle panels) using anti-HA-HRP and anti-HA antibodies, respectively. AnxA2-HA was localized at the plasma membrane of hepatocytes, albeit at varying levels, and was co-localized with LDLR at the basolateral surface (Figure 5C). Western blotting experiments show that in the liver of WT mice infected with Ad-A2, LDLR levels were increased on average by ∼50% compared to those infected with the empty Ad-Ctl (Figure 5A). Immunohistochemical analyses also revealed that LDLR levels were apparently increased in the liver of WT mice infected with Ad-A2, and that this increase is mostly localized at the hepatic cell surface (Figure 5C). In *Pcsk9* ^−/−^ mice, the immunostaining of hepatic LDLR appeared very strong as compared to WT livers, as previously reported [7], [9]. However, injection of Ad-Ctl or of Ad-A2 to *Pcsk9* ^−/−^ mice did not modify the level of LDLR, as seen by Western blot and immunocytochemistry (Figure 5B,C), emphasizing the PCSK9-specificity of the Ad-A2 effect in WT mice.

In order to detect changes in circulating LDLc, plasma lipoproteins of Ad-Ctl and Ad-A2 injected mice were separated by FPLC and their cholesterol content quantitated. Compared to WT mice, total circulating cholesterol was ∼40% lower in *Pcsk9^−/−^* mice, as previously reported [7], [9]. However, plasma FPLC profiles of Ad-A2 injected WT or *Pcsk9^−/−^* mice did not show significant changes in the levels of circulating LDLc compared to Ad-Ctl (*not shown*). These data indicate that in mice the overexpression of AnxA2 in liver by Ad-A2 may not be high enough in all hepatocytes to affect the levels of circulating LDLc.

### A Human AnxA2 Single Nucleotide Polymorphism (SNP) Correlates with Low Circulating PCSK9

The *in vivo* role of AnxA2 was assessed by sequencing AnxA2 exons 4, 5, 6 (coding for the R1 domain that binds PCSK9) from 43 individuals that were not receiving any medication and from 31 subjects taking statins (Table S2). This revealed a coding SNP variant, rs17845226, changing a Val to a Leu (V98L), which leads to a modified version of the R1 domain of AnxA2. Sequenced individuals were found in the following proportion for the V98L polymorphism; 10/43 were heterozygotes and 1/43 was homozygote in normal subjects and 5/31 were heterozygotes and 1/31 was homozygote in the hyperlipidemic group analyzed (Table S2). Other reported AnxA2 missense coding SNPs (dbSNP, http://www.ncbi.nlm.nih.gov/snp) occurring in R1 domain e.g. rs75993598 (E43K), rs11553794 (R63S), rs147297902 (A90V) or rs144035126 (T97M) were not found in our samples and probably represent rarer SNPs.

The analyses of plasma samples of healthy individuals who are carriers of the V98L variant revealed a significant (∼30%) reduction in circulating PCSK9 without changes in total cholesterol or LDLc levels (Table S2A). However, the individual exhibiting a homozygous form of the variant has >50% reduced levels of circulating PCSK9, >30% lower LDLc and >20% lower total cholesterol (Table S2A). While as expected, the mean plasma PCSK9 levels were ∼30% higher in statin treated subjects, in this group they were not significantly different between non-carriers and AnxA2 V98L carriers (Table S2B). This may be rationalized due to the dominant effect of statins in increasing plasma levels of PCSK9 [14], which could mask the effect of the heterozygote V98L mutation. Of note is the striking similarity between the healthy and statin-treated V98L homozygotes (Table S2), as both individuals had >50% lower plasma levels of PCSK9. Given the low number of subjects, especially the homozygote ones, these results remain preliminary and should be confirmed and replicated in a larger cohort. However, the data suggest that AnxA2 V98L SNP could be a GOF mutation, possibly allowing AnxA2 to bind more strongly to PCSK9, or allowing a better translocation to the cell surface where it could bind and inhibit PCSK9. Cell transfection and co-immunoprecipitation experiments demonstrate that AnxA2 V98L binds and inhibits PCSK9 at least as well as the AnxA2 WT form (Figure S5A,B). Further experiments specifically testing the binding affinity of AnxA2 V98L to PCSK9 and its inhibition by quantifying LDLR activity at the cell surface will be needed to define if the V98L mutation modifies the function of PCSK9.

## Discussion

The discovery of PCSK9 and its genetic relation to hypercholesterolemia [4], [6] led to a very exciting and active period of identification of the mechanisms of action of PCSK9 [15], [73], [74], and to the development of powerful animal genetic models [7], [9]. This has reached maturity to the point that we can entertain various viable therapeutic options. These include PCSK9 mAbs that disrupt the PCSK9≡LDLR interaction [52], and mRNA silencing strategies including siRNAs [47] or locked nucleic acid [48] approaches, all of which are in clinical trials [75]. The mAb approach has also targeted the CHRD domain of PCSK9 and revealed that the best mAb reduces by only 50% the PCSK9-dependent inhibitory effects on LDL uptake, without affecting the PCSK9≡LDLR interaction [62]. Our previous studies revealed that the R1 domain of AnxA2 can specifically bind the CHRD of PCSK9 and inhibit the function of this protein on LDLR, representing the first example of a natural inhibitor of PCSK9 activity [54]. However, since the expression of AnxA2 is not abundant in liver, the major source of PCSK9 (Figure S2), the physiological significance of this observation remained obscure. In this context, it was also observed that injection of PCSK9 in the bloodstream of mice spared the LDLR in a number of tissues, but was very active in selectively reducing the hepatic levels of this receptor [63]. Furthermore, transgenic mice overexpressing PCSK9 in hepatocytes [9] or kidney [67] also had little effect on LDLR levels in a number of extrahepatic tissues. This suggested that the mechanism for PCSK9 induced LDLR degradation might either lack a specific regulator in these tissues, or that PCSK9 function may be inhibited therein. Because AnxA2 is the only known natural inhibitor of PCSK9, we decided to test its possible regulation of PCSK9 function by analyzing the consequences of its genetic deletion. We therefore used *AnxA2^−/−^* mice to test the implication of AnxA2 in PCSK9 biology in extrahepatic tissues.

Analysis of the plasma of *AnxA2^−/−^* mice revealed higher levels of circulating PCSK9 (∼2-fold) and of LDLc (∼1.4-fold) without affecting HDLc (Table 1, Figure 1, and Figure S1). This suggested that lack of AnxA2 may increase the circulating levels of PCSK9 and/or its local bioavailability, and consequently its activity on LDLR. We next focused on the measurement of LDLR levels in various tissues of WT and *AnxA2^−/−^* mice. QPCR analysis of the mRNA levels of HMG-CoA reductase, PCSK9, LDLR and SREBP2 did not show significant variations between WT or *AnxA2^−/−^* mice in adrenal, colon or ileum (*not shown*). However, at the protein level, hepatic LDLR levels were decreased by ∼20% in the absence of AnxA2, but the reduction was much more marked in adrenals (∼50%) and colon (∼40%) (Figures 2,3), tissues known to be rich sources of AnxA2 (Figure S2), and resistant to PCSK9 effect [9], [63], [67]. This might be attributed to both increased levels of circulating PCSK9 as well as possibly enhanced activity of endogenous intestinal PCSK9 (Figure S2) due to the absence of its inhibitor AnxA2 in knockout mice. Why is extrahepatic LDLR protected from PCSK9-induced degradation? In adrenals, cholesterol is the building block for the synthesis of glucocorticoids and mineralocorticoids and its regulation is tightly controlled [76]. In mouse adrenals, cholesterol is mostly obtained from circulating HDL *via* the SR-BI receptor [76]. Since the adrenal steroid hormone production is normal in mice lacking LDLR, the role of the latter and its lack of regulation by PCSK9 in presence of AnxA2 are yet to be better elucidated. However, in humans, LDLR is also important for cholesterol uptake by adrenals in the acute phase of steroidogenesis and seems to be a major receptor that provides the cholesterol needed for steroid hormone production [77], [78]. How does AnxA2 regulate the functional activity of the PCSK9≡LDLR complex? It is possible that by binding to the CHRD (44), AnxA2 induces a conformational change in PCSK9 such that its interaction with the LDLR, and/or its cellular internalization is compromised. This allosteric model is supported by the observation that extracellular mAbs to the CHRD can also inhibit, albeit up to 50%, PCSK9-induced LDLR degradation without affecting the PCSK9≡LDLR interaction [62]. This emphasizes the importance of the CHRD in regulating the PCSK9 function on the LDLR internalization and degradation. However, it seems that these mAbs do not compete with AnxA2 in inhibiting PCSK9 function [62]. In our study, the peptides mimicking AnxA2 R1 domain directly inhibit the PCSK9≡LDLR interaction (Figure 4). Thus, modulating the CHRD function at multiple sites can reduce the PCSK9≡LDLR interaction and/or LDLR degradation. It was recently proposed that the prosegment of PCSK9 could interact with the CHRD within the ER and favors its secretion [79] and may thus affect the R1 AnxA2-CHRD interaction. However, the recent crystal structure [80] of the soluble extracellular ectodomain of LDLR in complex with PCSK9 did not show any interaction between the prosegment in mature PCSK9 and the CHRD at neutral pH. Therefore the role of the prosegment in regulating the extracellular PCSK9-AnxA2 interaction is not yet clear.

None of the published therapeutic anti-PCSK9 approaches used a small molecule inhibitor, possibly due to the relative flatness of the surface of interaction of the PCSK9≡LDLR complex [12], [15], [80]. Since we had already shown that the R1 domain of AnxA2 is the critical segment interacting with the CHRD, we further investigated the functional structural determinants within this domain using a far Western approach (Figure S4). The data revealed a complex interaction between AnxA2 and PCSK9 implicating many residues within the stretch of aa 25–108 of AnxA2, and emphasising the importance of exposed charged residues (Figure S4C,D). Since this domain spans 84 aa, and in view of the difficulty of synthesizing large peptides, we decided to synthesize a 73 aa peptide spanning aa 25–97, which represents the sequence least similar to the non-interacting AnxA1 (Figure S4A). Using a binding assay of the ectodomain of the LDLR to PCSK9, we found that this 73 aa peptide can indeed compete with the LDLR for PCSK9 binding with an IC_50_ of 0.6 µM (Figure 4). The potency of inhibition was reduced by ∼20-fold with the intermediate aa 49–97 peptide, and practically lost with a shorter one (aa 73–97), or upon mutating the positive charges ^77^ RRTKK ^81^ to ^77^AATAA^81^ in the C-terminal segment (Figure 4). Therefore, aa 25–97 of AnxA2 represent the first model peptide (beyond the LDLR-EGFA domain itself [23]) that can significantly inhibit PCSK9≡LDLR interaction. Future studies may refine this peptide and result in a more stable and potent derivative.

Even though AnxA2 is hardly expressed in liver, it is possible that its overexpression in this tissue may have an effect on the PCSK9 activity therein. A similar strategy was recently reported using the E3-ubiquitin ligase known as Idol, which also enhances LDLR degradation in extrahepatic tissues but is not expressed in liver [81]. We therefore expressed full length AnxA2 in the liver of WT and control *Pcsk9^−/−^* mice using an adenoviral recombinant. The data show that ectopic expression of AnxA2 in liver results in a ∼50% increase in total LDLR levels, as observed by Western blot and immunohistochemistry (Figure 5), without significantly affecting circulating LDLc (*not shown*). However, only very high levels of PCSK9 can reduce LDLR levels to the point that circulating LDLc are decreased, as observed in transgenic lines [9], and following injection of high doses of PCSK9 [63]. Thus, it is likely that the hepatic 50% LDLR increase in WT mice injected with Ad-A2, resulting in a variable AnxA2 expression in hepatocytes (Figure 5), may not be sufficient to cause a significant decrease in circulating LDLc. Therefore, a more efficient overexpression system that induces high expression of AnxA2 in all hepatocytes may be needed to lower LDLc levels, or the co-expression of its p11 partner, expressed at very low levels in the liver [82], may be required for more effective translocation of the cytosolic AnxA2 to the cell surface [56]. Nevertheless, the observed significant ∼1.5-fold increase in LDLR levels is not seen in *Pcsk9^−/−^* mice, attesting to the specificity of the AnxA2 effect, with the caveat that this could also relate to an already maximal response of LDLR expression in absence of PCSK9. Injection of a peptide mimetic of the R1 domain, which would bypass the need for cell surface translocation of AnxA2, may represent another approach to regulate PCSK9 function. Finally, transgenic mice overexpressing PCSK9 in an *AnxA2^−/−^* background may represent a good model for enhanced PCSK9 extrahepatic functions.

The data obtained in mice led us to predict that if a functional polymorphic variant is found in the R1 domain of AnxA2 it may affect circulating PCSK9 levels. By exon sequencing of the R1 domain exclusively, in subjects without any PCSK9 mutation, we identified a V98L polymorphism associated with low levels of PCSK9, but none associated with high levels of PCSK9. We found 15 heterozygotes and 2 homozygotes for the V98L modification (Table S2). On average, the two identified subjects with homozygote V98L variation had ∼50% lower levels of PCSK9 than heterozygotyes (Table S2). Although a clear trend is apparent for the association between the V98L variant of AnxA2 and lower circulating PCSK9 levels, more patients need to be screened for AnxA2 mutations before definitive conclusions can be reached on the protective properties of these possible GOF mutations and their relationship to statin response.

In conclusion, the present report demonstrates an extrahepatic physiological function of AnxA2 in regulating PCSK9's ability to enhance the degradation of the LDLR, especially in adrenals and the digestive organs. Whether AnxA2 is implicated in the fine regulation of PCSK9 function during embryonic development or in some situations requiring high levels of cholesterol, such as in regenerating tissues, will need further studies. The ability of an AnxA2 peptide to inhibit PCSK9 function may be a prelude to the synthesis of novel small molecule inhibitors of PCSK9.

## Supporting Information

Figure S1 **FPLC fractionation and lipoprotein cholesterol distribution of plasma of** ***AnxA2*** **^−/−^ mice.** Pooled plasma samples from 3 WT or 3 *AnxA2^−/−^* mice were fractionated by FPLC gel filtration using a Superose-6 column into very low-density lipoprotein (VLDL; fractions 15–21), intermediate- and low-density lipoprotein (IDL/LDL; fractions 22–36) and high-density lipoprotein (HDL; fractions 37–55). Cholesterol levels of fractions were determined by enzymatic assay. Comparison of the cholesterol content of each lipoprotein peak revealed a specific increase of LDLc in *AnxA2^−/−^* mice.
(PDF)

Figure S2 **Relative mRNA expression of** ***Ldlr*** **,** ***Pcsk9*** **and** ***AnxA2*** **in mouse tissues.** RNA samples were isolated from mouse tissues and quantitative polymerase chain reactions were performed using specific oligonucleotides for mouse *Ldlr*, *AnxA2* and *Pcsk9* and normalized to 10^6^ S16 mRNA levels, as described in Materials and Methods. Asterisks emphasise tissues that were analysed by WB and IHC for LDLR protein expression in this study.
(PDF)

Figure S3 **Intracellular co-localization of AnxA2 and PCSK9.** HepG2 cells transiently transfected with AnxA2 were incubated with conditioned medium from HEK293 cells overexpressing PCSK9-V5 for 60 min and then fixed and permeabilized. Cells were then incubated with anti-AnxA2 and anti-V5 antibodies and antibodies bound to their antigens were revealed with species-specific Alexa-647- (blue) and Alexa-555- (red) conjugated secondary antibodies, respectively. Arrows point to intracellular compartments where AnxA2 and PCSK9-V5 are co-localized. Bar = 10 µm.
(PDF)

Figure S4 **Fine mapping of the interacting sequence of AnxA2 R1 domain to PCSK9.** (A) Primary sequence alignment of human AnxA2 (aa 25–108) and AnxA1 (aa 34–117). PCSK9-interacting sequence (aa 34–108) of AnxA2 is highlighted in green with emphasis on critical residues as determined by far Western blotting (FWB) (shown in red). (B) For FWB, HEK293 cells were transfected with full-length human HA-tagged AnxA2 (FL) or deletants thereof (Δ25–36, Δ37–48, Δ49–61, Δ62–75, Δ37–66, Δ74–88, Δ82–88, Δ89–101, Δ102–108). Following SDS-PAGE (10%) of cell lysates, proteins were transferred on nitrocellulose membranes and incubated with conditioned media obtained from HEK293 cells overexpressing human V5-tagged PCSK9. Bound PCSK9-V5 was detected using a V5-HRP antibody. Expression of the AnxA2-HA constructs was verified on separate membranes by Western Blotting (WB) using an anti-HA-HRP antibody. (C) Superposition of R1 domain structures of porcine AnxA1 (PDB 1MCX; blue) and human AnxA2 (PDB 1W7B; gray) were generated using the Pymol Molecular Graphics System. (D) HEK293 cells were transfected with full-length HA-tagged AnxA2 (FL) or HA-tagged AnxA2 mutants harbouring selected residues of AnxA1 and analyzed by FWB as describe above. The arrow point to the specific binding of PCSK9-V5 to AnxA2-HA constructs and the asterisk mark a non-specific band present in all lanes. These data are representative of three separate experiments.
(PDF)

Figure S5 **AnxA2 V98L variant co-immunoprecipitate with PCSK9 and reduces LDLR degradation.** (A) CHO-K1 cells were co-transfected with PCSK9-V5 and either with an empty pIRES-V5 vector (Mock), HA-tagged AnxA2 WT or HA-tagged AnxA2 V98L variant. PCSK9-V5 was immunoprecipitated using an anti-V5 antibody (IP∶V5) and its interaction with AnxA2 was probed by Western blot using an anti-HA antibody (WB∶HA). Controls of PCSK9-V5 immunoprecipitation (IP∶V5, WB∶V5) and of plasmid overexpression in cell lysates (WB∶HA or WB∶V5) are also shown. (B) Western blot for LDLR in whole-cell lysates from HepG2 cells that were either mock transfected or transfected with HA-tagged AnxA2 WT or HA-tagged AnxA2 V98L. Equal protein loading and overexpression of plasmids were demonstrated with anti-β-actin and anti-HA antibodies.
(PDF)

Table S1 **Oligonucleotides used for site-directed mutagenesis of AnxA2.**
(PDF)

Table S2 **Physical characteristics, fasting plasma lipids, and PCSK9 levels of 43 healthy volunteers (A) and 31 hypercholesterolemicsubjects treated with statins (B) based on the V98L genotype.**
(PDF)
