# Supplementary figures and images for: Annexin A2 Is a Natural Extrahepatic Inhibitor of the PCSK9-Induced LDL Receptor Degradation
Source: PLoS One. 2012 Jul 27;7(7):e41865. doi: 10.1371/journal.pone.0041865 (PMC3407131; doi:10.1371/journal.pone.0041865)

Supplemental Figure S1

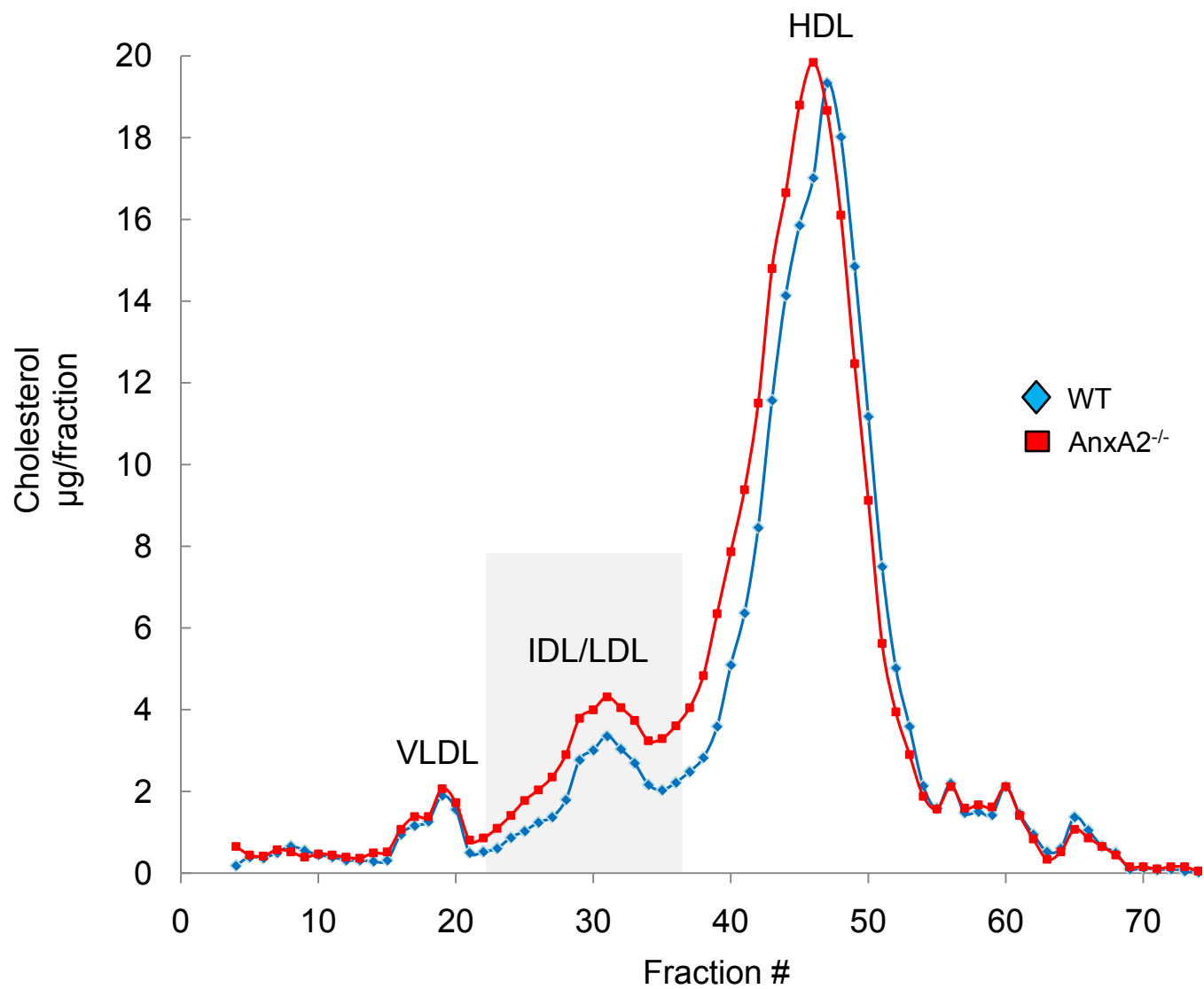

Supplement: Figure S1 — FPLC fractionation and lipoprotein cholesterol distribution of plasma of AnxA2 −/− mice. Pooled plasma samples from 3 WT or 3 AnxA2−/− mice were fractionated by FPLC gel filtration using a Superose-6 column into very low-density lipoprotein (VLDL; fractions 15–21), intermediate- and low-density lipoprotein (IDL/LDL; fractions 22–36) and high-density lipoprotein (HDL; fractions 37–55). Cholesterol levels of fractions were determined by enzymatic assay. Comparison of the cholesterol content of each lipoprotein peak revealed a specific increase of LDLc in AnxA2−/− mice. (PDF) [file pone.0041865.s001.pdf]

# Supplemental Figure S2

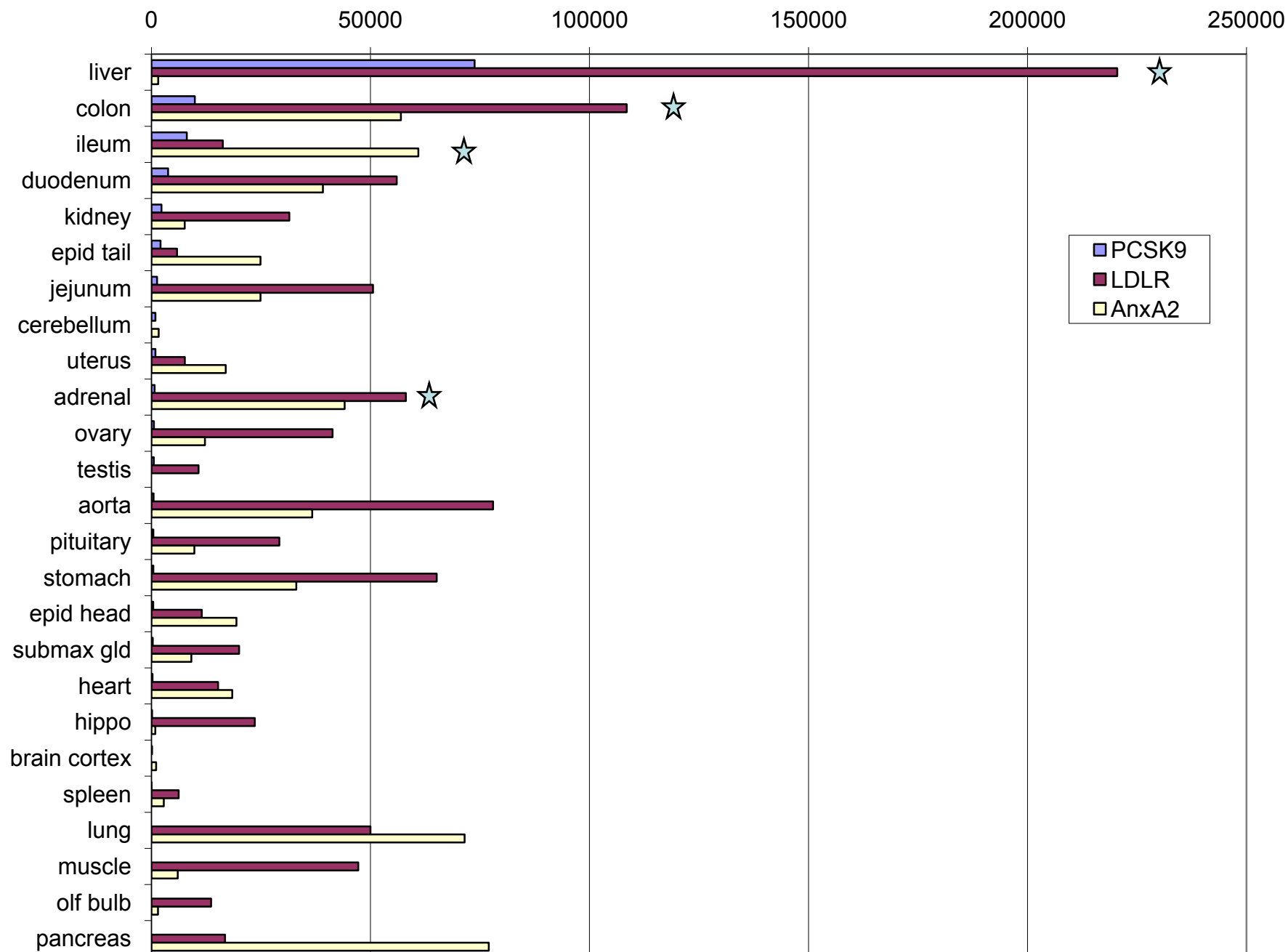

Supplement: Figure S2 — Relative mRNA expression of Ldlr , Pcsk9 and AnxA2 in mouse tissues. RNA samples were isolated from mouse tissues and quantitative polymerase chain reactions were performed using specific oligonucleotides for mouse Ldlr, AnxA2 and Pcsk9 and normalized to 106 S16 mRNA levels, as described in Materials and Methods. Asterisks emphasise tissues that were analysed by WB and IHC for LDLR protein expression in this study. (PDF) [file pone.0041865.s002.pdf]

Supplemental Figure S3

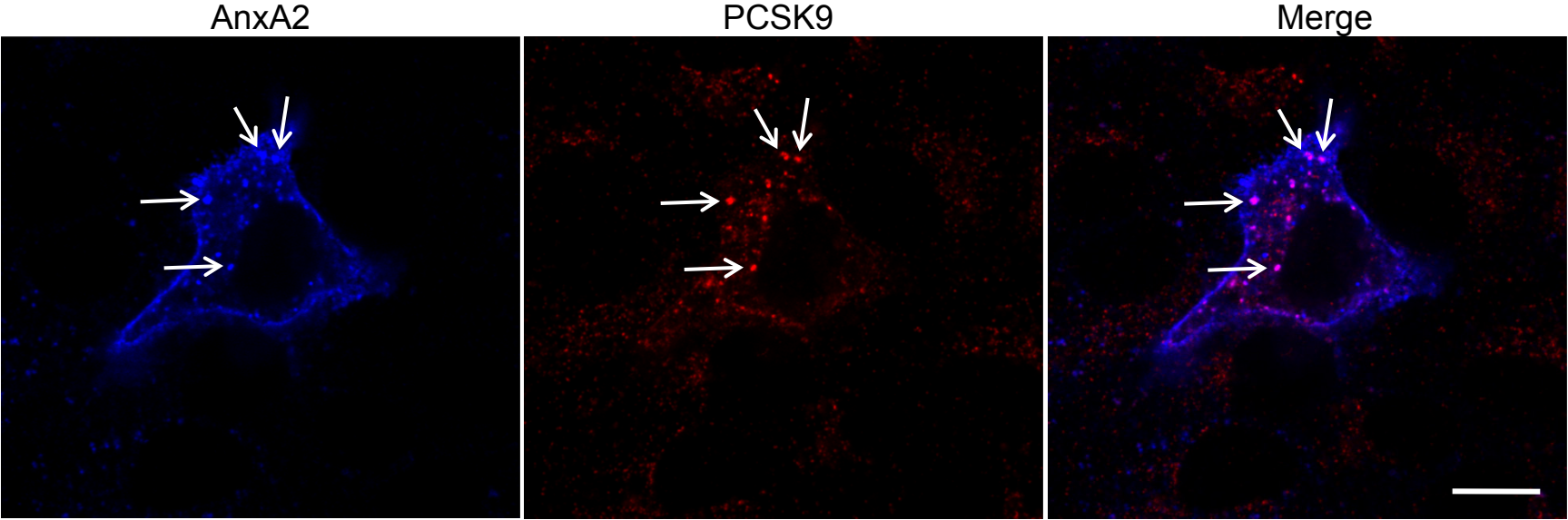

Supplement: Figure S3 — Intracellular co-localization of AnxA2 and PCSK9. HepG2 cells transiently transfected with AnxA2 were incubated with conditioned medium from HEK293 cells overexpressing PCSK9-V5 for 60 min and then fixed and permeabilized. Cells were then incubated with anti-AnxA2 and anti-V5 antibodies and antibodies bound to their antigens were revealed with species-specific Alexa-647- (blue) and Alexa-555- (red) conjugated secondary antibodies, respectively. Arrows point to intracellular compartments where AnxA2 and PCSK9-V5 are co-localized. Bar = 10 µm. (PDF) [file pone.0041865.s003.pdf]

Supplemental Figure S5

A

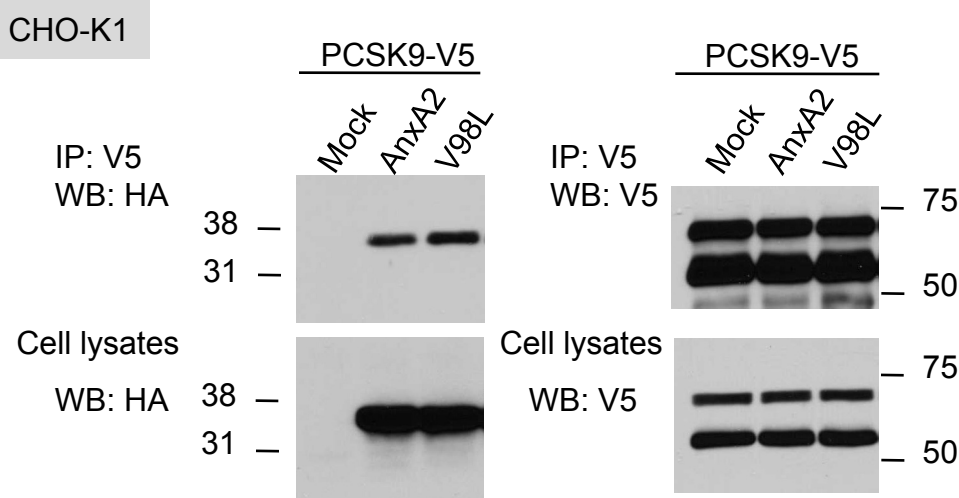

B

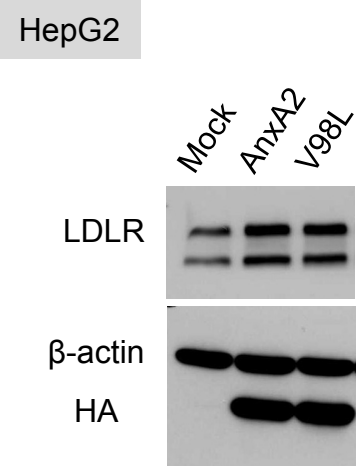

Supplement: Figure S5 — AnxA2 V98L variant co-immunoprecipitate with PCSK9 and reduces LDLR degradation. (A) CHO-K1 cells were co-transfected with PCSK9-V5 and either with an empty pIRES-V5 vector (Mock), HA-tagged AnxA2 WT or HA-tagged AnxA2 V98L variant. PCSK9-V5 was immunoprecipitated using an anti-V5 antibody (IP∶V5) and its interaction with AnxA2 was probed by Western blot using an anti-HA antibody (WB∶HA). Controls of PCSK9-V5 immunoprecipitation (IP∶V5, WB∶V5) and of plasmid overexpression in cell lysates (WB∶HA or WB∶V5) are also shown. (B) Western blot for LDLR in whole-cell lysates from HepG2 cells that were either mock transfected or transfected with HA-tagged AnxA2 WT or HA-tagged AnxA2 V98L. Equal protein loading and overexpression of plasmids were demonstrated with anti-β-actin and anti-HA antibodies. (PDF) [file pone.0041865.s005.pdf]
