# Supplementary material for: Annexin A2 Is a Natural Extrahepatic Inhibitor of the PCSK9-Induced LDL Receptor Degradation
Source: PLoS One. 2012 Jul 27;7(7):e41865. doi: 10.1371/journal.pone.0041865 (PMC3407131; doi:10.1371/journal.pone.0041865)
Supplement: Figure S4 — Fine mapping of the interacting sequence of AnxA2 R1 domain to PCSK9. (A) Primary sequence alignment of human AnxA2 (aa 25–108) and AnxA1 (aa 34–117). PCSK9-interacting sequence (aa 34–108) of AnxA2 is highlighted in green with emphasis on critical residues as determined by far Western blotting (FWB) (shown in red). (B) For FWB, HEK293 cells were transfected with full-length human HA-tagged AnxA2 (FL) or deletants thereof (Δ25–36, Δ37–48, Δ49–61, Δ62–75, Δ37–66, Δ74–88, Δ82–88, Δ89–101, Δ102–108). Following SDS-PAGE (10%) of cell lysates, proteins were transferred on nitrocellulose membranes and incubated with conditioned media obtained from HEK293 cells overexpressing human V5-tagged PCSK9. Bound PCSK9-V5 was detected using a V5-HRP antibody. Expression of the AnxA2-HA constructs was verified on separate membranes by Western Blotting (WB) using an anti-HA-HRP antibody. (C) Superposition of R1 domain structures of porcine AnxA1 (PDB 1MCX; blue) and human AnxA2 (PDB 1W7B; gray) were generated using the Pymol Molecular Graphics System. (D) HEK293 cells were transfected with full-length HA-tagged AnxA2 (FL) or HA-tagged AnxA2 mutants harbouring selected residues of AnxA1 and analyzed by FWB as describe above. The arrow point to the specific binding of PCSK9-V5 to AnxA2-HA constructs and the asterisk mark a non-specific band present in all lanes. These data are representative of three separate experiments. (PDF) [file pone.0041865.s004.pdf]

Supplemental Figure S4

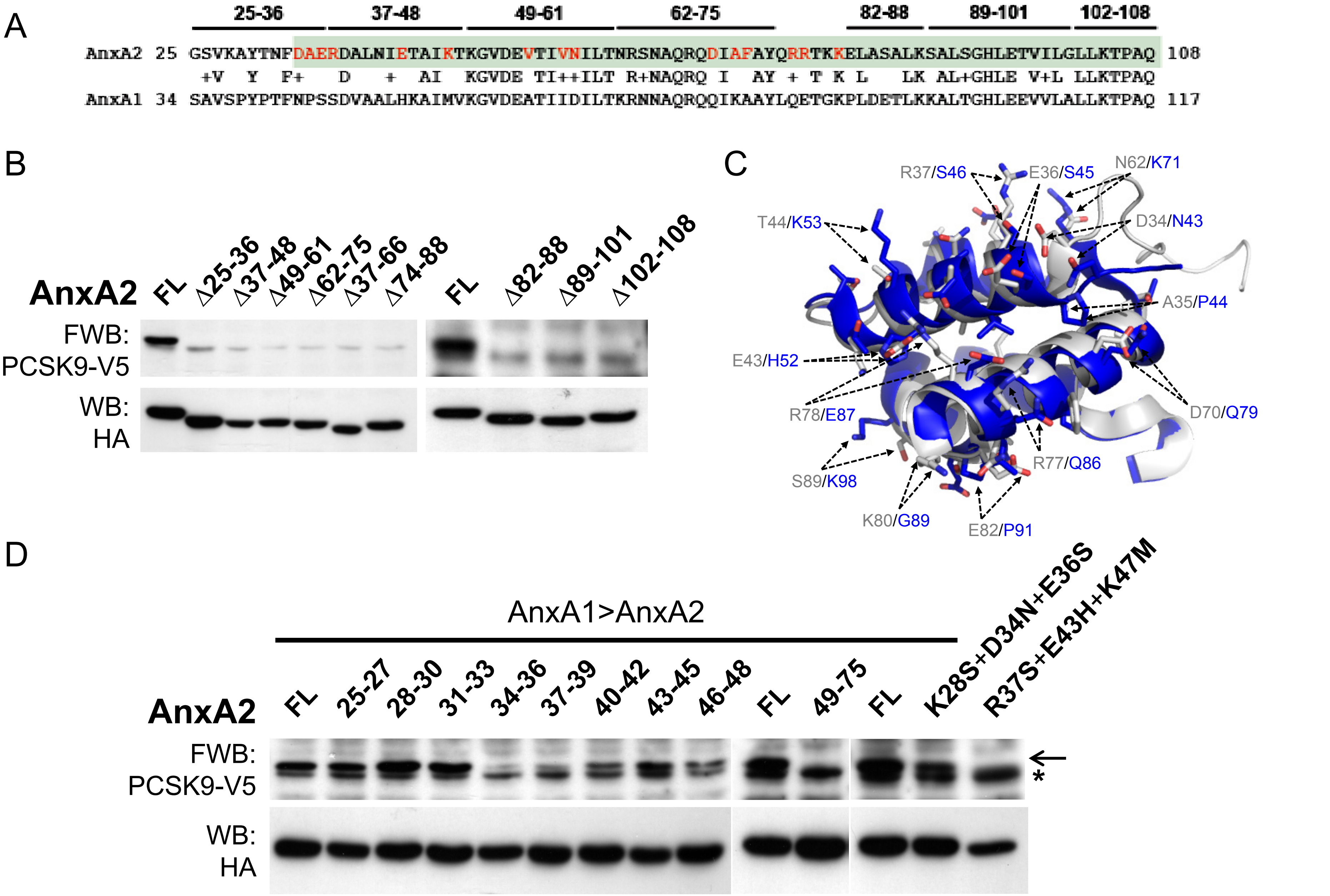

Seidah et al.,
