# Supplementary material for: Annexin A2 Is a Natural Extrahepatic Inhibitor of the PCSK9-Induced LDL Receptor Degradation
Source: PLoS One. 2012 Jul 27;7(7):e41865. doi: 10.1371/journal.pone.0041865 (PMC3407131; doi:10.1371/journal.pone.0041865)
Supplement: Table S1 — Oligonucleotides used for site-directed mutagenesis of AnxA2. (PDF) [file pone.0041865.s006.pdf]

Table S1. Oligonucleotides used for site-directed mutagenesis of AnxA2.

Mutants	Sense	Antisense
Δ25-36	5'-CCAAGTGCATATCGGGATGCTTTGAACATTGAAAC-3'	5'-CATCCCGATATGCACTTGGGGGTGT-3'
Δ37-48	5'-GATGCTGAGAAAGGTGTGGATGAGGTCAC-3'	5'-CCACACCTTTCTCAGCATCAAAGTTAGTATAGGC-3'
Δ49-61	5'-CATCAAGACCAACCGCAGCAATGCAC-3'	5'-CTGCGGTTGGTCTTGATGGCTGTTTCAATG-3'
Δ62-75	5'-CAACATTTTGACCAGAAGGACCAAAAAGGAACTTGC-3'	5'-GGTCCTTCTGGTCAAAATGTTGACAATGGTG-3'
Δ37-66	5'-GATGCTGAGCAGAGACAGGATATTGCCTTC-3'	5'-CCTGTCTCTGCTCAGCATCAAAGTTAGTATAGGC-3'
Δ74-88	5'-GCCTTCTCAGCCTTATCTGGCCAC-3'	5'-CCAGATAAGGCTGAGAAGGCAATATCCTGTCTC-3'
Δ82-88	5'-GAAGGACCAAAAAGTCAGCCTTATCTGGCCAC-3'	5'-GATAAGGCTGACTTTTTGGTCCTTCTCTGGTAGG-3'
Δ89-101	5'-CAGCACTGAAGCTATTGAAGACACCTGCTCAG-3'	5'-GTCTTCAATAGCTTCAGTGCTGATGCAAGTTC-3'
Δ102-108	5'-GATTTTGGGCTATGACGCTTCTGAGCTAAAAG-3'	5'-GAAGCGTCATAGCCCAAAATCACCGTCTC-3'
> 25-27 (AnxA1)	5'-CTACACCCCCAAGTGCATATAGCGCTGTC-3'	5'-GACAGCGCTATATGCACTTGGGGGTGTAG-3'
> 28-30 (AnxA1)	5'-GGGTCTGTCAGTCCCTATACTAACTTTG-3'	5'-CAAAGTTAGTATAGGGACTGACAGACCC-3'
> 31-33 (AnxA1)	5'-GTCAAAGCCTATCCTACCTTTGATGCTGAG-3'	5'-CTCAGCATCAAAGGTAGGATAGGCTTTGAC-3'
> 34-36 (AnxA1)	5'-CTATACTAACTTTAATCCTAGTCGGGATGCTTTG-3'	5'-CAAAGCATCCCGACTAGGATTAAAGTTAGTATAG-3'
> 37-39 (AnxA1)	5'-CTTTGATGCTGAGAGCGATGTTTTGAACATTG-3'	5'-CAATGTTCAAAACATCGCTCTCAGCATCAAAG-3'
> 40-42 (AnxA1)	5'-CGGGATGCTGCGGCCCTTGAAACAGCCATC-3'	5'-GATGGCTGTTTCAAGGGCCGCAGCATCCCG-3'
> 43-45 (AnxA1)	5'-CTTTGAACATTCACAAAGCCATCAAGACC-3'	5'-GGTCTTGATGGCTTTGTGAATGTTCAAAG-3'
> 46-48 (AnxA1)	5'-GAAACAGCCATCATGGTCAAAGGTGTGG-3'	5'-CCACACCTTTGACCATGATGGCTGTTTC-3'
> 49-75 (AnxA1)	5'-AACAATGCACAGAGACAGCAAATTAAAGCCGCCTACCAGAGAAGGACCAAAAAGGAACTTG-3'	5'-CTGGTAGGCGGCTTTAATTTGCTGTCTCTGTGCATTGTTGCGTTTGGTCAAAATGTCG-3'
> 49-61 (AnxA1)	5'-GGCCACCATTATCGACATTTTGACCAACCGCAGCAATGCACAGAG-3'	5'-CAAAATGTCGATAATGGTGGCCTCATCCACACCTTTGGTC-3'
> 62-75 (AnxA1)	5'-AAACGCAACAATGCACAGAGACAGCAGATTAAGGCCGCCTACCAGAGAAGGACC-3'	5'-GGCCTTAATCTGCTGTCTCTGTGCATTGTTGCGTTTGGTCAAAATGTTGACAATGGTG-3'
K28S+D34N+E36S	5'-TCAGCCTATACTAACTTTAATGCTTCGCGGGATGCTTTGAACATTG-3'	5'-CGAAGCATTAAAGTTAGTATAGGCTGAGACAGACCCATATGCACTTG-3'
R37S+E43H+K47M	5'-CGGATGCTTTGAACATTCACACAGCCATCATGACCAAAGGTGTGGATGAG-3'	5'-CATGATGGCTGTGTGAATGTTCAAAGCATCCGACTCAGCATCAAAGTTAGTATAGGC-3'
V98L	5'-GGAGACGCTGATTTTGGGCCTATTG-3'	5'-CAATAGGCCCAAAATCAGCGTCTCC-3'

Seidah et al.,
