# Supplementary material for: Annexin A2 Is a Natural Extrahepatic Inhibitor of the PCSK9-Induced LDL Receptor Degradation
Source: PLoS One. 2012 Jul 27;7(7):e41865. doi: 10.1371/journal.pone.0041865 (PMC3407131; doi:10.1371/journal.pone.0041865)
Supplement: Table S2 — Physical characteristics, fasting plasma lipids, and PCSK9 levels of 43 healthy volunteers (A) and 31 hypercholesterolemicsubjects treated with statins (B) based on the V98L genotype. (PDF) [file pone.0041865.s007.pdf]

Table S2. Physical characteristics, fasting plasma lipids, and PCSK9 levels of 43 healthy volunteers (A) and 31 hypercholesterolemic subjects treated with statins (B) based on the V98L genotype. (mean ± SD. P-values were obtained using a two-tailed Student's t-test. *, p=0.01. **, p=0.02. ***, p=0,001).

A

	AnxA2 V98L Non-carriers (min–max) n = 32	AnxA2 V98L heterozygotes (min–max) n = 10	AnxA2 V98L homozygotes (n = 1)
Age (years)	43 ± 15 (21–76)	45 ± 14 (25–67)	23
BMI (kg/m^2)	25.1 ± 3.9 (19.5–35.2)	23.7 ± 2.8 (18.9–28.1)	20.6
PCSK9 (ng/ml)	80.4 ± 42.2 (25.9–190.2)	*56.1 ± 20.9 (39.5–96.8)	38.0
TC (mmol/L)	5.37 ± 0.82 (3.82–7.02)	5.66 ± 1.79 (3.01-8.43)	3.79
TG (mmol/L)	1.37 ± 0.89 (0.47–3.74)	1.43 ± 0.71 (0.45–2.65)	0.53
LDLc (mmol/L)	3.11 ± 084 (1.76–5.21)	3.34 ± 1.43 (1.17–4.83)	1.94
HDLc (mmol/L)	1.62 ± 0.45 (0.87–2.58)	1.49 ± 0.29 (1.08–1.99)	1.61

B

	AnxA2 V98L Non-carriers (min–max) n = 25	AnxA2 V98L heterozygotes (min–max) n = 5	AnxA2 V98L homozygotes (n = 1)
Age (years)	55 ± 17 (19–86)	65 ± 18 (40–82)	59
BMI (kg/m^2)	26.0 ± 3.9 (19.5–33.1)	27.1 ± 5.8 (19.9–33.1)	24.4
PCSK9 (ng/ml)	106.4 ± 37.8 (36.5–165.4)	112.1 ± 12.3 (97.7–123.6)	41.5
TC (mmol/L)	4.93 ± 0.95 (3.26–7.72)	**4.47 ± 0.25 (4.08–4.71)	3.77
TG (mmol/L)	1.61 ± 1.20 (0.54–5.65)	1.67 ± 0.88 (0.93–3.00)	1.35
LDLc (mmol/L)	2.89 ± 0.74 (1.62–4.76)	***2.27 ± 0.18 (2.06–2.48)	2.06
HDLc (mmol/L)	1.35 ± 0.38 (0.85–2.16)	1.38 ± 0.53 (0.97–2.20)	1.09

Seidah et al.,
